# Diminished androgen levels are linked to irritable bowel syndrome and cause bowel dysfunction in mice

**DOI:** 10.1172/JCI150789

**Published:** 2022-01-18

**Authors:** Daniella Rastelli, Ariel Robinson, Valentina N. Lagomarsino, Lynley T. Matthews, Rafla Hassan, Kristina Perez, William Dan, Peter D. Yim, Madison Mixer, Aleksandra Prochera, Amy Shepherd, Liang Sun, Kathryn Hall, Sarah Ballou, Anthony Lembo, Judy Nee, Meenakshi Rao

**Affiliations:** 1 Department of Pediatrics, Boston Children’s Hospital and Harvard Medical School, Boston, Massachusetts, USA.; 2 Department of Pediatrics, Columbia University Medical Center, New York, New York, USA.; 3 Department of Gastroenterology, Beth Israel Deaconess Medical Center, Boston, Massachusetts, USA.; 4 Department of Anesthesiology, Columbia University Medical Center, New York, New York, USA.; 5 The Manton Center for Orphan Disease Research, Boston Children’s Hospital, Boston, Massachusetts, USA.; 6 Division of Preventive Medicine, Brigham and Women’s Hospital, Boston, Massachusetts, USA.

**Keywords:** Gastroenterology, Neuroscience, Neuroendocrine regulation, Sex hormones

## Abstract

Functional gastrointestinal disorders (FGIDs) have prominent sex differences in incidence, symptoms, and treatment response that are not well understood. Androgens are steroid hormones present at much higher levels in males than females and could be involved in these differences. In adults with irritable bowel syndrome (IBS), a FGID that affects 5% to 10% of the population worldwide, we found that free testosterone levels were lower than those in healthy controls and inversely correlated with symptom severity. To determine how this diminished androgen signaling could contribute to bowel dysfunction, we depleted gonadal androgens in adult mice and found that this caused a profound deficit in gastrointestinal transit. Restoring a single androgen hormone was sufficient to rescue this deficit, suggesting that circulating androgens are essential for normal bowel motility in vivo. To determine the site of action, we probed androgen receptor expression in the intestine and discovered, unexpectedly, that a large subset of enteric neurons became androgen-responsive upon puberty. Androgen signaling to these neurons was required for normal colonic motility in adult mice. Taken together, these observations establish a role for gonadal androgens in the neural regulation of bowel function and link altered androgen levels with a common digestive disorder.

## Graphical abstract

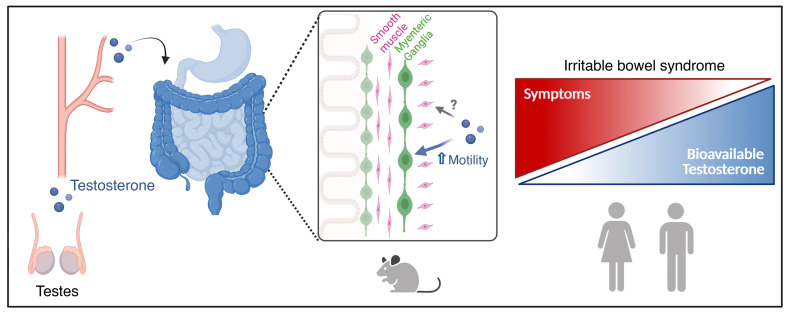

## Introduction

Almost all disorders of the gut-brain axis, from inflammatory bowel disease to neurological disorders with GI comorbidities, exhibit sex differences (1–3). Irritable bowel syndrome (IBS) is one of the most common of these disorders and is characterized by abdominal pain and altered stool patterns (diarrhea, constipation, or both). IBS is less common in men than women in Western countries, so many studies to date have focused on estrogen signaling in IBS pathophysiology (4, 5). Much less attention has been paid to androgens, such as testosterone, which circulate at higher levels in postpubertal males than females. They could also be involved, possibly with protective effects. Isolated studies have probed this possibility in small cohorts of patients with IBS or a related functional gastrointestinal disorder (FGID) and found conflicting associations between testosterone levels and symptoms (6–8). The role of androgens in these disorders thus remains unclear.

Androgens signal through a common androgen receptor (AR), a ligand-dependent transcription factor, to modulate gene expression. In addition to this canonical pathway, androgens can also have nongenomic effects that are faster and transcription independent (9). Testosterone and 5α-dihydrotestosterone (DHT) are the primary ligands for AR, but DHT is 10-fold more potent, and unlike testosterone, cannot be aromatized into estradiol. Radiolabeled DHT binds cell nuclei throughout the GI tract of nonhuman primates, predominantly in the tunica muscularis (10). This suggests that intestinal smooth muscle is androgen-responsive, similar to vascular smooth muscle. Consistent with this possibility, intestinal muscle strips isolated from mice exhibit increased contractility when preincubated with androgens in vitro (11, 12). The significance of this signaling in regulating bowel function in vivo, however, is unknown.

## Results

### IBS symptom severity is linked to diminished androgens.

**Figure 1:**
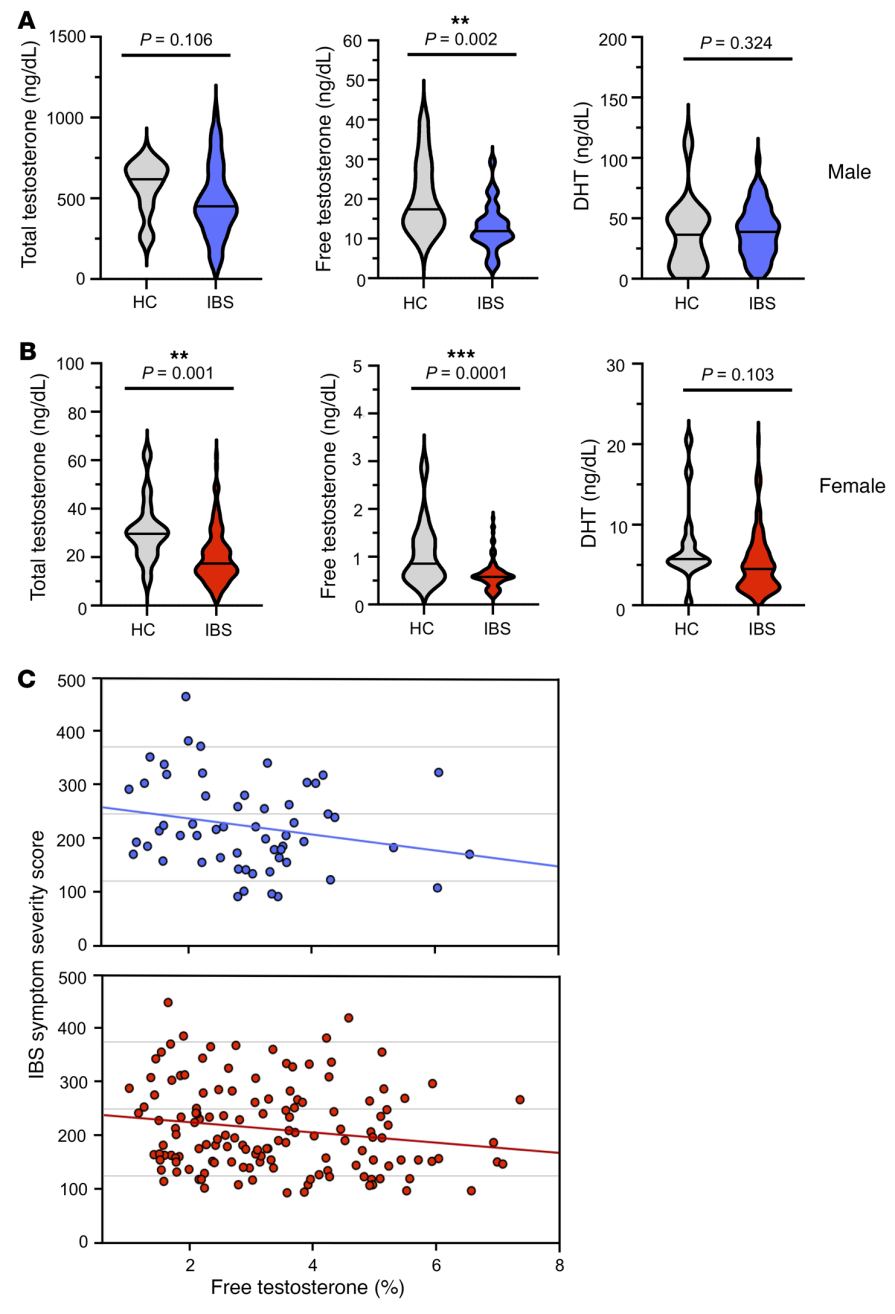
Diminished testosterone levels are linked to the diagnosis and severity of irritable bowel syndrome. (**A **and** B**) Violin plots show serum levels of total testosterone, free testosterone, and dihydrotestosterone (DHT) in postpubertal males (**A**) and females (**B**) with IBS (*n =* 58 males, 150 females) and healthy controls (HC; *n =* 14 males, 14 females). Free testosterone indicates the proportion of total testosterone that was measured as free. Median value is indicated as bar in center of each plot. *P* values are for unpaired *t* tests comparing means of log-transformed values between IBS and HC groups. (**C**) Scatter plots illustrating the relationship between IBS-SSS and percentage of free testosterone in the serum of male (blue) and female (red) patients with IBS. Higher score indicates more severe symptoms. Percentage of free testosterone was no different between males and females (*t* test, *P* = 0.180), and so these groups were combined for analysis. Partial correlation analyses controlling for age and sex revealed a negative association between percentage of free testosterone and IBS symptom severity (*r* = –0.19, *P* = 0.009). **P < 0.005, ***P < 0.0005.

To better determine whether androgen signaling could play a role in FGIDs, we used a highly sensitive mass spectrometry assay to measure total testosterone, percentage of free testosterone, and DHT levels in serum from 208 adults with IBS at the baseline visit of a randomized controlled trial (Clinical Trials.gov NCT02802241; study design in ref. 13 and participant details in Supplemental Table 1; supplemental material available online with this article; https://doi.org/10.1172/JCI150789DS1). We found that both males and females with IBS had lower levels of circulating free testosterone than healthy controls, with no difference in DHT levels (Figure 1, A and B and Supplemental Table 2), suggesting that IBS was associated with selective androgen deficits.

To ascertain whether androgen levels were linked to the degree of symptoms that patients with IBS experienced, we examined the association between free testosterone and IBS symptom severity score (IBS-SSS), a commonly used composite measure of IBS severity (14). IBS-SSS inversely correlated with the percentage of free testosterone levels in both sexes (Figure 1C), indicating that having a lower proportion of bioavailable testosterone was associated with more severe IBS symptoms. Because we retrospectively profiled a cohort assembled to test a therapy, our study had a limited number of healthy controls. Nevertheless, we found that both the diagnosis and severity of IBS were linked to lower levels of bioavailable testosterone, raising the possibility that diminished androgen signaling causes bowel dysfunction.

### Gonadal androgens are required for normal gastrointestinal motility.

**Figure 2:**
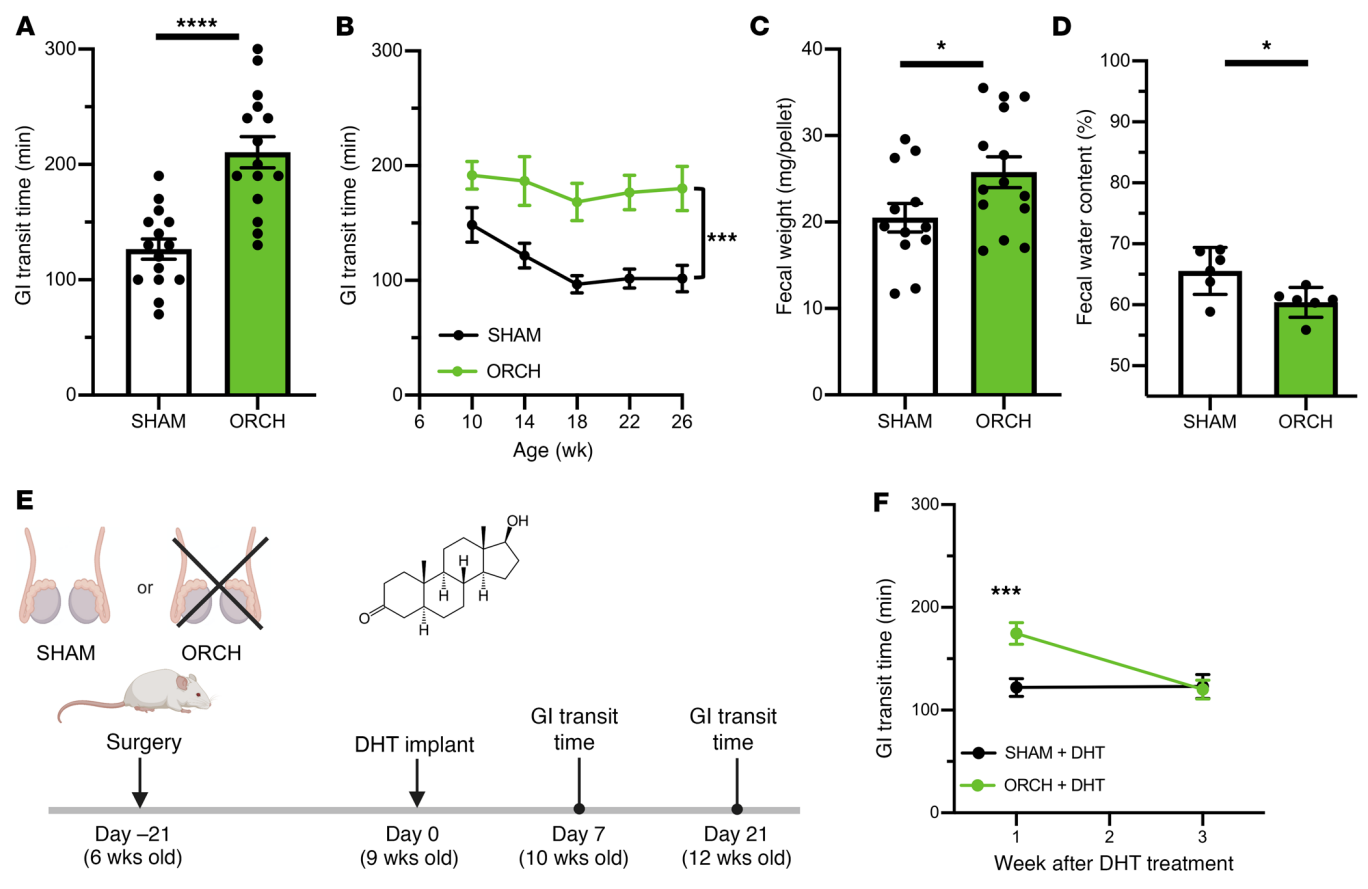
Gonadal androgens are required for normal gastrointestinal transit in vivo. (**A**) Total GI transit time was measured in 10-week-old wildtype (FVB/NJ strain) male mice 4 weeks after sham surgery (*n =* 15) or bilateral orchiectomy (*n =* 15). ORCH mice had markedly delayed GI transit compared with SHAM mice (*P* < 0.0001). (**B**) Total GI transit time was measured every 4 weeks after surgery and remained consistently slower in ORCH mice compared with SHAM controls (*n =* 6/group, *P* = 0.001, 2-way repeated measures ANOVA). (**C**) Average fecal pellet mass was greater in ORCH mice than SHAM controls (*n* =12–14/group; *P* = 0.0433). (**D**) Fecal water content was lower in ORCH mice than SHAM controls (*n =* 6/group; *P* = 0.0198). (**E**) Schematic of experimental design for androgen rescue experiment in **F**. Bilateral orchiectomy or sham surgeries were performed on 6-week-old FVB/NJ mice. Subcutaneous pellets that continuously released DHT, a nonaromatizable androgen, were implanted into mice from both groups 3 weeks later. Total GI transit time was then measured at 10 and 12 weeks of age. (**F**) Three weeks of DHT supplementation resulted in the normalization of GI transit time in ORCH mice to that of SHAM mice (*P* = 0.0029, 2-way repeated measures ANOVA; *n =* 10–11/group). *P < 0.05, ***P < 0.005, and ****P < 0.001. Unpaired *t* tests were used to compare pairs of group means in **A**, **C**, and **D**. Error bars reflect standard error of the mean.

To determine how diminished androgen signaling could cause bowel dysfunction and IBS symptoms, we depleted gonadal androgens in wildtype male mice by performing bilateral orchiectomy shortly after puberty (experimental group hereafter referred to as ORCH). Four weeks later, GI transit time in ORCH mice was 40% slower than that in littermate controls who underwent sham operations (SHAM mice; Figure 2A). Remarkably, this motility deficit was fixed and persistent, without any improvement over 16 weeks (Figure 2B). ORCH mice had 25% larger fecal pellets with less water content (Figure 2, C and D), additional features of chronic constipation. These observations indicate that gonadal function is necessary for normal GI motility in male mice and that its loss causes constipation.

The testes are the predominant source of circulating androgens in males but also elaborate other bioactive molecules. To ascertain to what extent androgen deficiency was selectively responsible for slowed GI transit in ORCH mice, we restored androgens 3 weeks after surgery by implanting a subcutaneous pellet in each mouse that continuously eluted 0.125 mg DHT per day (Figure 2E). Supplemental DHT did not affect GI transit time in SHAM mice but completely normalized the GI transit deficit in ORCH mice within 3 weeks (Figure 2F), suggesting that a single androgen was sufficient to rescue the dysmotility caused by orchiectomy.

### Gonadal androgen deficiency selectively disrupts colonic motility.

**Figure 3:**
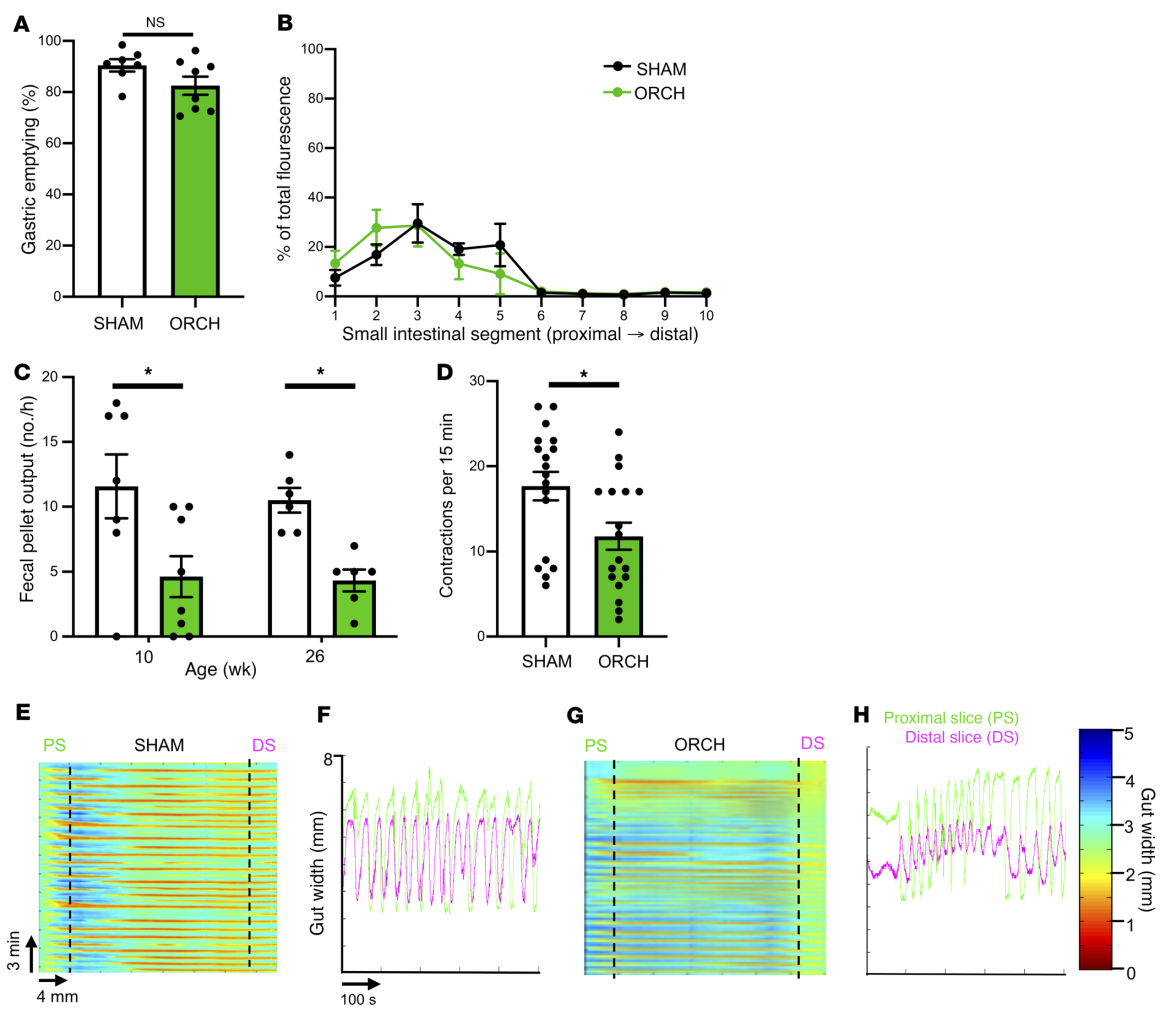
Gonadal androgen deficiency selectively disrupts colonic motility. (**A**) Gastric emptying of a nonabsorbable dye was no different between SHAM and ORCH mice (*n =* 7–8/group; *P* = 0.0967). (**B**) Small intestinal transit, measured as the distance traveled by a fluorescent dye along the small intestine 15 minutes after gavage, was no different between SHAM and ORCH mice. The *x* axis represents intestinal segment number (proximal → distal) (*n =* 7–8 mice/group; each animal’s small intestine was divided into 10 equal segments for measurement of fluorescence). (**C**) Number of fecal pellets spontaneously expelled over 1 hour, measured 4 weeks after surgery (10 weeks of age) and 20 weeks after surgery (26 weeks of age) in SHAM and ORCH mice (*n =* 6–8/group; 10 weeks, *P* = 0.0111; 26 weeks, *P* = 0.0456). *P < 0.05. (**D**) Ex vivo imaging showed that colons from ORCH mice had fewer effective, colonic migrating motor contractions (defined as those starting proximally and propagating > 50% of the length of the bowel), than those from SHAM mice (*P = 0.0150). (**E**–**H**) Representative spatiotemporal maps illustrating contractile activity of colons from SHAM (**E**) and ORCH (**G**) mice ex vivo. Color reflects degree of gut contraction (measured as gut width). Vertical slice analysis at proximal (PS) and distal segments (DS) of the SHAM mouse colon (**F**) shows regular periodic contractions that start proximally and propagate distally (each green drop in gut width is followed by a pink drop). Similar analysis of ORCH mouse colon (**H**) shows more disorganized contractions with less efficient propagation. See Supplemental Video 1 for representative videos. Unpaired *t* tests were used to compare pairs of group means in **A**, **C**, and **D**. Error bars reflect standard error of the mean.

GI motility represents a complex repertoire of motor behaviors that vary along the digestive tract. To determine which motor behaviors were androgen dependent, we analyzed gastric, small intestinal, and colonic motility in ORCH mice. Gastric emptying and small bowel transit were unchanged in ORCH mice (Figure 3, A and B). One-hour fecal pellet output, however, was 60% lower in ORCH mice (Figure 3C), suggesting that their motility deficit localized to the colon. To characterize how androgen deficiency altered colonic motor behaviors, we studied them ex vivo in full-length colonic segments isolated from SHAM and ORCH mice 4 or more weeks after surgery. The neural circuits required for colonic peristalsis are part of the enteric nervous system (ENS) intrinsic to the GI tract (15). As such, rhythmic colonic migrating motor contractions (CMMCs) that propel luminal contents in the expected oral-to-anal direction can be observed ex vivo (16, 17). We found that colons from ORCH mice exhibited highly disorganized contraction patterns (see Supplemental Video 1) with fewer effective CMMCs that propagated at least 50% of the length of the bowel (Figure 3, D–H). The velocity, duration, and amplitude of the CMMCs that did propagate effectively remained intact (Supplemental Figure 1). These observations indicate that loss of gonadal function in male mice selectively disrupts colonic motility, with gut intrinsic effects on the organization of motor behavior.

### Androgen deficiency does not alter gut smooth muscle function.

**Figure 4:**
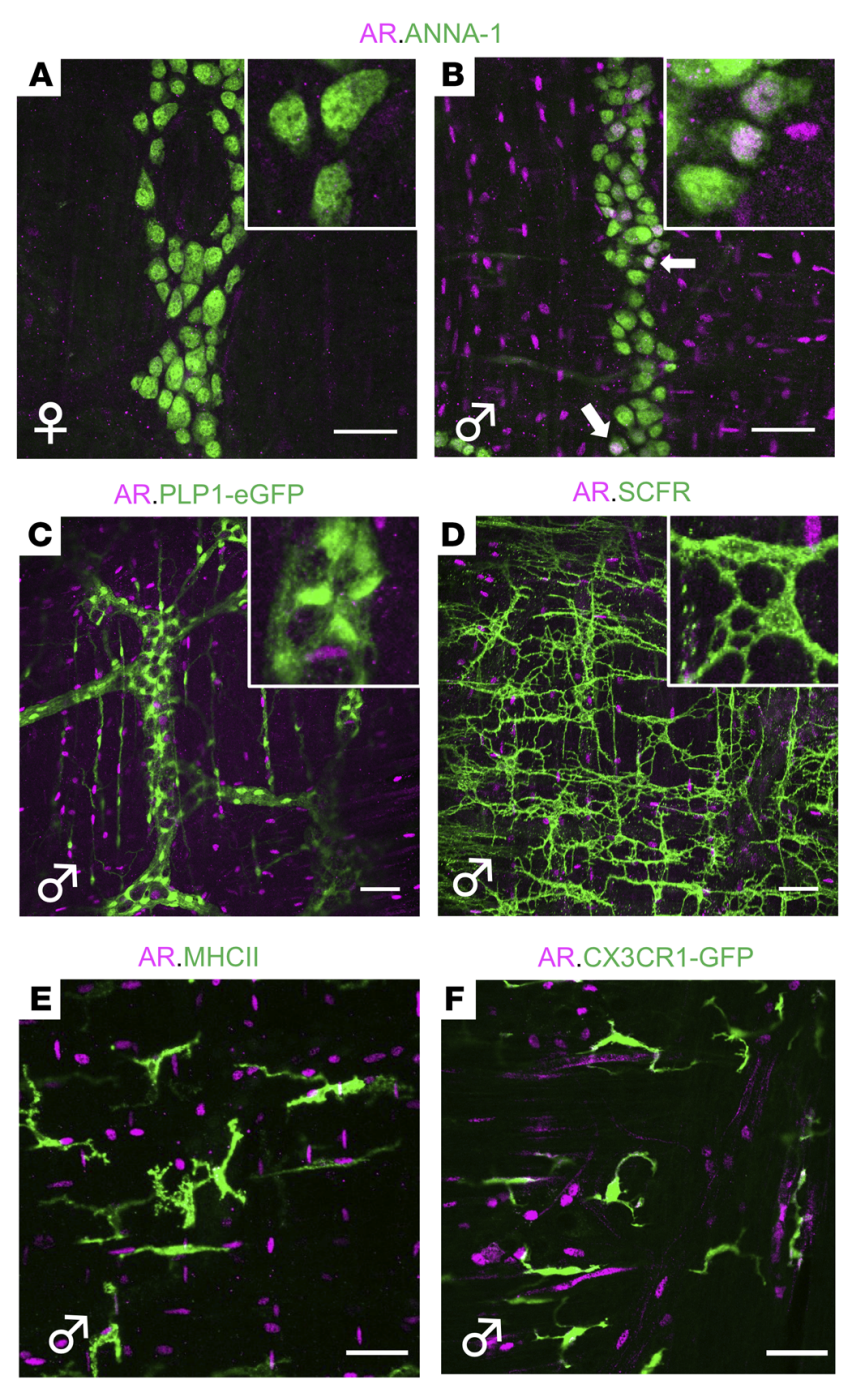
Androgen receptor expression in mouse colon. (**A** and** B**) AR immunoreactivity is undetectable in the muscularis externa of the colon from an adult female mouse (**A**), but abundant in that of an adult male (**B**). The majority of AR-expressing cells are in the smooth muscle syncytium but a subset colocalizes with the pan-neuronal marker (ANNA-1; arrows) in the myenteric plexus. (**C**) Enteric glia labeled with green fluorescence protein (GFP) in the colon of an adult male PLP1-eGFP mouse do not express AR (45). (**D**) Interstitial cells of Cajal labeled by SCFR immunoreactivity do not express AR in the adult male mouse colon. Scale bars in **A**–**D** = 50 μm. Insets in **A**–**D** show representative cells from associated images at higher magnification. (**E**) Muscularis macrophages labeled by MHCII immunoreactivity do not express AR in the adult male mouse colon. (**F**) Muscularis macrophages labeled with GFP in the colon of adult male CX3CR1^GFP/+^ mouse do not express AR. Scale bars **E **and** F** = 40 μm. All images are representative of observations made in a minimum of 3 mice per condition.

Normal colonic motility arises from the coordinated actions of many cell types, including cells of the smooth muscle syncytium, interstitial cells of Cajal (ICCs), enteric glia, muscularis macrophages, and enteric neurons, all found within the muscularis externa (18–21). To determine which of these might mediate the effects of androgens on colonic motility, we examined AR expression in detail. Surprisingly, while *Ar* transcripts were expressed in the colons of adult mice of both sexes (Supplemental Figure 2), AR protein was found by immunohistochemical staining only in the male muscularis externa (Figure 4, A and B), presumably in the smooth muscle syncytium, consistent with previous reports (10, 12, 22). Unexpectedly, AR was also present in the myenteric plexus of the male ENS, where it colocalized with the pan-neuronal soma marker ANNA-1 (Figure 4B). Remarkably, 17% ± 1.7% of neurons within the myenteric plexus, but none in the submucosal plexus, expressed AR (mean ± SEM; *n =* 4 mice). AR was not detected in enteric glia, muscularis macrophages, or ICCs (Figure 4, C–F), and these cell populations appeared intact in ORCH mice (Supplemental Figure 3). In total, these observations suggest that both intestinal smooth muscle and a substantial proportion of enteric neurons respond to androgens in the adult male colon.

**Figure 5:**
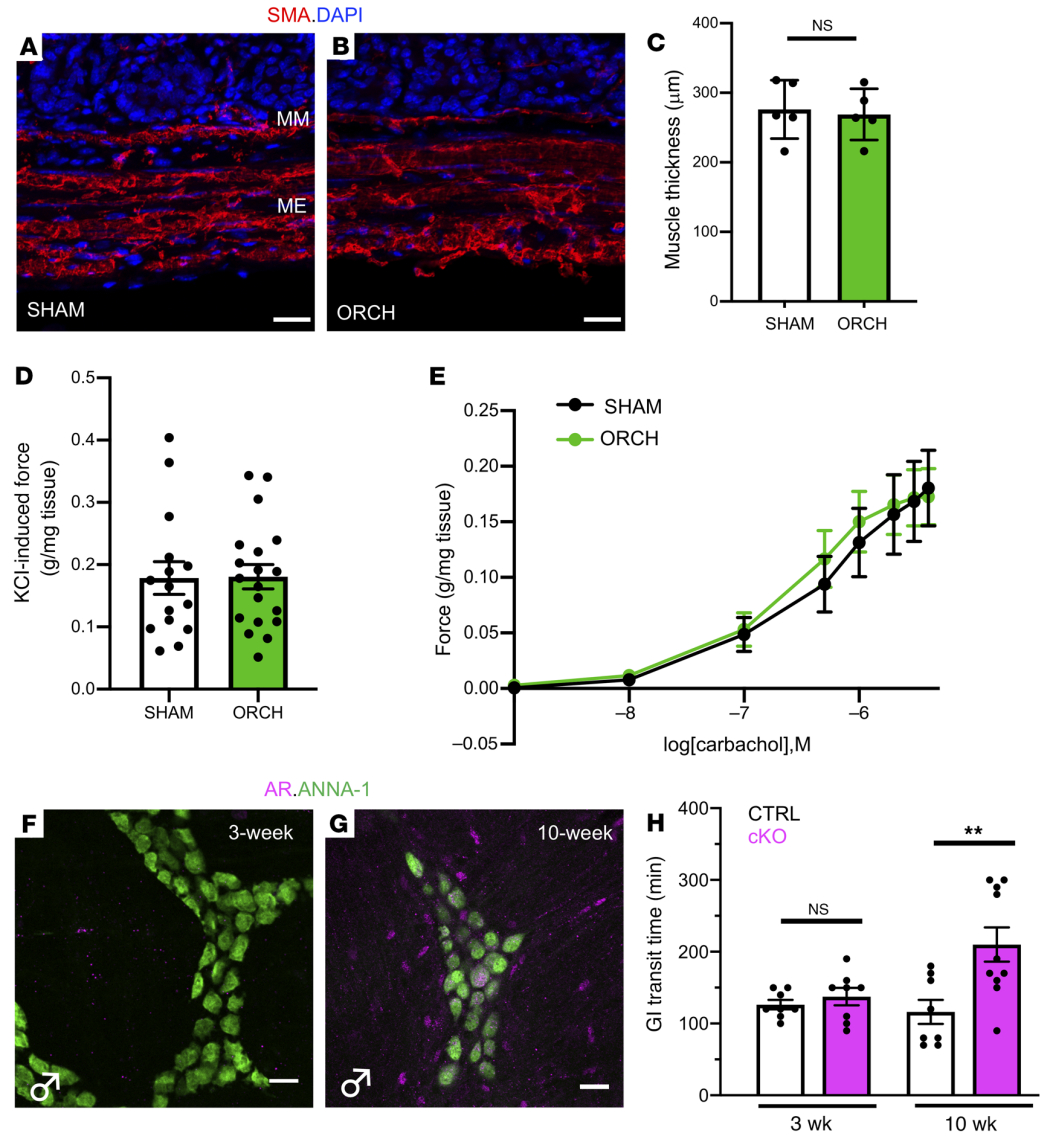
Androgen effects on gut motility are mediated by signaling in neurons, rather than smooth muscle. (**A** and **B**) Cross-sections of colons from SHAM and ORCH mice immunostained for α-smooth muscle actin (SMA) show that the muscularis externa (ME) and muscularis mucosa (MM) are grossly normal in ORCH mice. Cell nuclei marked with DAPI. Scale bars = 20 μm. Tissues for analyses in **A**–**E** were isolated from mice 5 to 7 weeks after surgery. (**C**) There is no difference in colonic smooth muscle thickness between SHAM and ORCH mice (*n =* 5 mice/group; *P* = 0.7824). (**D**) Maximum contractile force exerted by colonic segments from SHAM (*n =* 15 segments from 5 mice) and ORCH mice (*n =* 19 segments from 6 mice) is no different (*P* = 0.9503). Force measurements in **D** and **E** were normalized to tissue mass. (**E**) The dose-dependent contractile force generated by colonic segments upon exposure to the acetylcholine receptor agonist carbachol is no different between segments isolated from SHAM and ORCH mice (*n =* 15–19, as in **D**). (**F** and **G**) AR immunoreactivity is undetectable in the muscularis externa of a colon from a 3-week-old wildtype male mouse (**F**). By the postpubertal age of 10 weeks, robust AR expression is observed both within a subset of enteric neurons and in the smooth muscle surrounding the myenteric plexus (**G**). Enteric neuronal soma are marked by ANNA-1 immunoreactivity. Scale bar = 50 μm. (**H**) GI transit time was no different in prepubertal (3-week-old) CTRL and cKO mice. When reassessed at a postpubertal age (10 weeks), GI transit time in cKO mice was prolonged (*n* = 8–10 mice/group; *P* = 0.0027). **P < 0.005. Unpaired *t* tests were used to compare pairs of group means in **C**, **D** and **H**. Error bars reflect standard error of the mean. Images in **A**, **B**, **F**, and **G** are representative of observations made in a minimum of 3 mice per condition.

Colonic dysmotility in ORCH mice could thus reflect a requirement for androgen signaling in intestinal smooth muscle, enteric neurons, or both. Smooth muscle bulk, measured by immunohistochemical staining for α-smooth muscle actin, was no different between SHAM and ORCH mice (Figure 5, A–C). To test smooth muscle function, we measured the contractility of acutely isolated colonic rings in the presence of tetrodotoxin, which blocks voltage-gated sodium channels and thus most fast synaptic transmission in the ENS (23). Both maximal contractile force of the smooth muscle, induced by KCl, and dose-dependent contractile force in response to the acetylcholine receptor agonist carbachol, were no different between SHAM and ORCH mice (Figure 5, D and E). These findings show that loss of male gonadal function did not diminish intestinal smooth muscle bulk or contractility and suggest that colonic dysmotility in ORCH mice is instead due to a requirement for androgen signaling in enteric neurons.

### Neuronal androgen signaling is required for normal GI motility.

Androgen effects on neurons have been most studied in the brain, where they influence circuits in 2 distinct phases in both mice and humans. During development, a transient surge of androgens produced by the testes establishes sex-specific circuits in certain brain regions, such as the hypothalamus (24). The second phase occurs upon puberty when androgens surge again in males and modulate the activity of these and other brain regions important for sex-specific behaviors (25). Consistent with this, circulating testosterone levels in male mice exhibit a biphasic pattern, with an initial peak within 24 hours of birth and a second much larger peak at 5 weeks of age, preceding breeding capability by 4 to 5 days (26). We found that AR expression was undetectable in the colons of prepubertal male mice (Figure 5F), but robust in both enteric neurons and surrounding cells in the muscularis externa by 7 weeks of age (Figure 4B and Figure 5G), suggesting that the ENS becomes androgen responsive upon puberty. Bilateral orchiectomy after puberty had no effect on neuronal density in the myenteric plexus (Supplemental Figure 3, C and D and Supplemental Figure 4D), implying that androgen deficiency affects the function of enteric neurons rather than their genesis or survival.

To determine if androgen signaling was necessary for enteric neuronal regulation of gut motility, we generated conditional knockout mice lacking AR in enteric neurons but not in the surrounding smooth muscle. Given the absence of Cre recombinase lines specific to enteric neurons, we used the Wnt1^Cre2^ transgenic line, which has been widely utilized to manipulate genes in neural crest derivatives, including the ENS (27, 28). After confirming that Cre activity in Wnt1^Cre2^ mice was detectable in the ENS but not in other cells in the muscularis externa (Supplemental Figure 5), we generated Wnt1^Cre2^Ar^fl/Y^ mice (hereafter AR^Wnt1KO^) that lacked AR expression in enteric neurons but not neighboring cells in the smooth muscle syncytium (Supplemental Figure 6). Three-week-old, prepubertal AR^Wnt1KO^ males had the same average GI transit time as their Ar^fl/Y^ littermate controls (Figure 5H). In contrast, 10-week-old postpubertal AR^Wnt1KO^ males had delayed GI transit that phenocopied ORCH mice (Figure 5H). Altogether, these findings indicate that the ENS becomes androgen responsive during male puberty and that androgen signaling through AR in the peripheral nervous system (PNS) is necessary for normal gut motility in adult mice.

**Figure 6:**
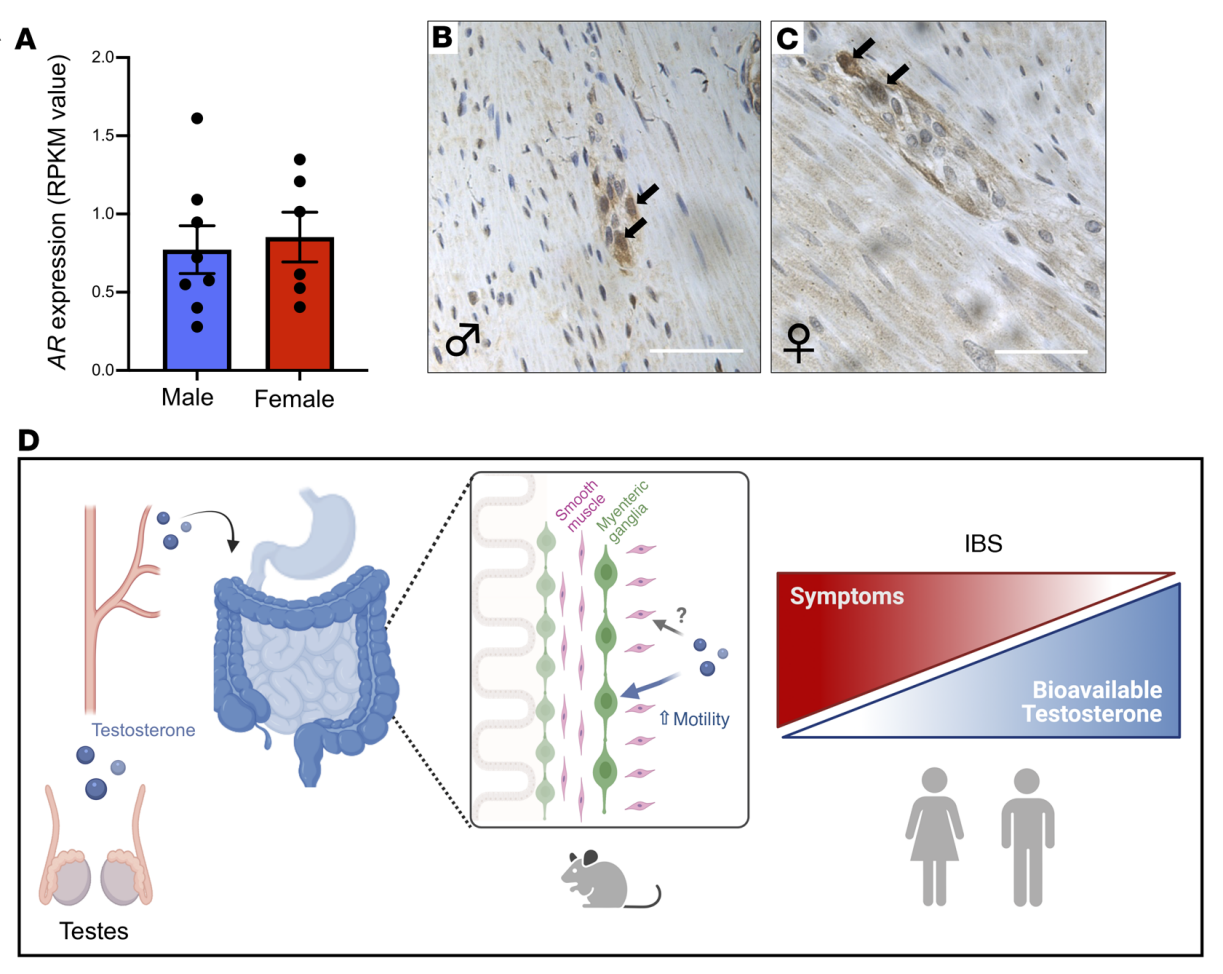
Androgen receptor is expressed in the human colonic ENS. (**A**) Analysis of *AR* expression in the transcriptomes of human colonic myenteric ganglia obtained by RNA-sequencing samples isolated by laser capture microdissection (29), reveals that *AR* is detectable at similar levels in male and female ganglia (*n =* 6–8 biological replicates shown with average RPKM value of 3 technical replicates each). (**B **and **C**) Cross sections of human colonic tissue show that AR immunoreactivity is evident in both males (**B**) and females (**C**) (*n =* tissue from 3 male and 3 female patients examined; representative images shown). The majority of AR-immunoreactive cells are located in the smooth muscle but a subset is in myenteric ganglia and exhibit cell soma and nuclear size typical of neurons (arrows). Scale bars in **B** and **C** = 10 μm. (**D**) Schematic of working model of androgen regulation of colonic motility and the potential link to IBS. Gonadal androgens signal to the smooth muscle syncytium and myenteric neurons in the postpubertal mouse colon. Androgen signaling to neurons, but not muscle, is essential for normal colonic motor behaviors in mice. In humans, lower androgen levels are associated with more severe IBS symptoms in males and females.

To ascertain if androgen signaling to enteric neurons could similarly play a role in human bowel function, we examined AR expression in the human ENS at the transcript and protein levels. Analysis of published RNA sequencing data from myenteric ganglia isolated by laser capture microdissection from the colons of healthy postpubertal adults (29) revealed *AR* transcript expression in male and female ganglia at comparable levels (Figure 6A). Immunohistochemical staining affirmed that AR was expressed in a subset of myenteric neurons in both sexes (Figure 6, B and C), indicating that the human ENS is capable of androgen response. These observations suggest that the molecular infrastructure enabling androgen-dependent regulation of colonic motility in mice is conserved in humans and may explain the link between diminished androgen levels and IBS symptom severity (Figure 6D).

## Discussion

Circulating androgen levels differ markedly in males and females after puberty and are further regulated at the local tissue level, offering a robust substrate for sex differences in health and disease. Here we show that diminished androgen levels are linked to the diagnosis and severity of IBS, a common FGID, highlighting the crucial need to study this group of hormones more widely in digestive disease. Patients with IBS are often classified as having diarrhea-predominant (IBS-D) or constipation-predominant (IBS-C) symptoms (30). While our retrospective study included patients of both subtypes, it was not sufficiently powered to enable comparisons between these groups. Future prospective studies that are powered to detect subtype-specific differences in androgen levels among patients with IBS and that incorporate objective measurements of colonic motility along with patient-reported symptoms would be valuable.

In seeking to learn how androgen deficits might give rise to IBS symptoms, we uncovered a mechanism by which gonadal androgen signaling to the ENS regulates gut motility in vivo (Figure 6D). The ENS has many different types of neurons in the myenteric plexus (28, 29, 31), almost all of which are involved in regulating gut motility in some capacity. Future work to determine which enteric neurons are androgen-responsive, how androgens modulate their activity, and the effects of this signaling on both motility and abdominal pain will yield new insights on GI biology and FGID pathophysiology. Previous studies of androgen signaling to the PNS largely focused on sex-specific motor functions, such as the penile erectile response (32). Our findings indicate that androgens have much broader homeostatic roles in modulating peripheral neuronal activity that merit exploration.

Like other steroids, circulating androgens are eliminated from the body by hepatic glucuronidation followed by excretion into urine and bile. Bile is secreted into the gut lumen where specific commensal microbes can deconjugate glucuronides, generating high local levels of steroid hormones, especially in the colon (33, 34). This phenomenon could constitute a rich source of ligands for AR-expressing neurons that innervate the gut and explain why colonic motility was particularly vulnerable to the effects of androgen deficiency in our study. Modulating peripheral nervous system function by recycling androgens into bioactive forms could be a mechanism by which microbes modulate host physiology.

Androgen deficiency as well as androgen excess have been linked to changes in the gut microbiome in both clinical and preclinical studies (35). Microbial dysbiosis could have contributed to the dysmotility observed in ORCH mice. Future studies that work to disentangle to what extent dysmotility drives dysbiosis and vice versa in androgen-deficient states will be informative. Recent work in rodent models of prostate cancer has shown that androgen deprivation positively selects for commensal gut microbes that synthesize androgens (36). Any such microbial compensation was apparently insufficient to rescue AR signaling to the ENS, however, because the dysmotility in ORCH mice persisted for several months without improvement.

Our preclinical studies focused on male mice because we did not detect AR expression in the female colon by immunohistochemical staining. However, the human tissue and IBS data suggest that androgen signaling is likely to be involved in gut functions in both sexes. This could reflect species-specific differences or, possibly, the limits of immunohistochemistry to detect low levels of AR in female mouse tissue. Consistent with this possibility, our RT-qPCR data as well as observations from another group using transgenic reporter mice show that *Ar* is transcriptionally active in the female mouse intestine (37). Even in males, however, we found that *Ar* transcript levels were not reflective of AR protein expression (Supplemental Figure 2). Taken together, these findings indicate that *Ar* undergoes tissue-specific, posttranscriptional regulation, and that the sex-specific roles of AR signaling in the bowel will require dedicated study.

Detailed examination of GI motility in ORCH mice revealed that androgen deficiency selectively altered colonic motility without any effects on gastric emptying or small intestinal transit, consistent with a previous study in rats that failed to find any effects of castration on upper GI motility (38). While we found that AR was expressed in the muscularis externa of both the stomach and colon in mice, in the ENS it was found exclusively in the colonic myenteric plexus. This selectivity likely explains why dysmotility was restricted to the colon in ORCH mice, and further supports the idea that AR signaling in neurons rather than smooth muscle is essential for normal regulation of gut motility. The functional significance of AR expression in the intestinal smooth muscle syncytium in vivo remains to be determined.

Androgen levels decline with age in at least a subset of men, and up to 20% of males over the age of 60 have low levels of bioavailable testosterone (39). Thus, diminished androgen signaling in the PNS may play a role not just in disease but also in the consequences of normal aging on gut functions. Colonic transit time and fecal water content drop dramatically with age in rodents (40, 41), and the prevalence of constipation increases with age in humans (42). Future studies manipulating androgen signaling in the context of aging could reveal to what extent androgen deficits and age-related constipation are linked.

Overall, our observations establish androgen-AR signaling as a critical pathway in the normal regulation of colonic motility and suggest that it should be considered in the pathophysiology of any disorder affecting the gut-brain axis. Emerging studies are revealing new roles for androgens in the gut, including dampening inflammatory responses in a specific subset of gastric lymphocytes (43) and modulating stromal cell effects on the small intestinal epithelium (44). These advances combine to suggest that androgen signaling is a complex, highly regulated pathway playing crucial homeostatic roles in the gut, and that these roles need to be broadly considered in human health as well as disease.

## Methods

Please see the Supplemental Material for detailed methods.

### Next-generation sequencing data.

AR expression in human colonic myenteric ganglia was analyzed using a publicly available bulk RNA-sequencing data set generated from samples obtained by laser capture microdissection (LCM; *n =* 6–8 samples per sex and 3 technical replicates per sample) (29). Raw data are available from National Center for Biotechnology Information Gene Expression Omnibus (GEO) under accession number GSE153202.

### Study approval.

All human subjects in the IRB-approved trial were recruited from a single center in the United States of America, and written informed consent was obtained from all subjects prior to their inclusion. Mice were handled per protocols approved by the IACUCs of Boston Children’s Hospital and Columbia University Medical Center.

## Author contributions

DR and MR conceived the study and wrote the initial manuscript. DR, AR, VNL, LTM, KP, AP, MM, WD, PDY, AS, and MR performed experiments and analyzed results. RH, SB, KH, and MR performed the analysis of the human sex hormone data. LS performed transcriptomic analysis. MR, KH, JN, and AL obtained funding for the studies. All authors revised and approved the final manuscript.

## Supplementary Material

Supplemental data

Supplemental video 1

## Version 1 11/30/2021

In-Press Preview

## Version 2 01/18/2022

Electronic publication
