# Supplementary material for: Diminished androgen levels are linked to irritable bowel syndrome and cause bowel dysfunction in mice
Source: J Clin Invest. 2022 Jan 18;132(2):e150789. doi: 10.1172/JCI150789 (PMC8759776; doi:10.1172/JCI150789)
Supplement: Supplemental data [file jci-132-150789-s202.pdf]

SUPPLEMENTAL MATERIAL

Materials and Methods

Mouse lines

Mice were housed in a specific pathogen-free facility with a 12-hour dark cycle and handled per protocols approved by the Institutional Animal Care and Use Committees of Boston Children's Hospital and Columbia University Medical Center, and adherent to the NIH Guide for the Care and Use of Laboratory Animals. PLP1^{eGFP} mice (JAX 033357; Jackson Laboratory, Bar Harbor, ME) were bred on a FVB/NJ (JAX 001800) background. CX3CR1$^{GFP/+}$ mice (JAX 005582) and all other mice were maintained on a C57Bl/6j background. Wnt1^{Cre2} hemizygous males (JAX 022137) were bred with female Rosa26$^{Ai9/Ai9}$ mice (JAX 007676) to generate Wnt1^{Cre2}::Rosa26$^{Ai9/+}$ mice to test Cre activity. AR$^{flx/flx}$ (EMMA 02579; Infrafrontier, Munich, Germany) female mice were bred with Wnt1^{Cre2} hemizygous males to generate Wnt1^{Cre2}::AR$^{flx/Y}$ mice and AR$^{flx/Y}$ littermate controls. Wnt1^{Cre2}::AR$^{flx/Y}$ mice were born at expected Mendelian ratios, had similar body weights to AR$^{flx/Y}$ littermates (Supplemental Figure 6), and were well-appearing.

Orchiectomy and sham surgical procedures

Six-week-old male FVB/NJ mice were anesthetized with 1-3% inhalable isoflurane (Patterson veterinary NDC 14043-704-05), administered ophthalmic eye ointment (Dechra NDC 17033-211-38), 5-10 mg/kg subcutaneous meloxicam (Putney NDC 26637-621-01), and subcutaneous bupivacaine at the incision site (MWI Veterinary Supply, Boise, ID; 0.25%, 2-3 mg/kg diluted with 50% saline for final 0.125% solution). Fur was removed from the scrotum, and the surgical area was sterilized with chlorhexidine and 70% ethanol. Using sterile technique, a single incision was made on the midline of the scrotum. Blunt forceps were used to grasp one testicular fat pad, and a ligature of absorbable suture material was placed around the epididymis. Using sharp scissors, the testicle was removed, and the

remaining tissue returned to the scrotal sac. Using the same incision, the procedure was repeated to excise the second testicle. After ensuring adequate bleeding control, the skin was closed with simple interrupted non-absorbable sutures. For sham operation, the same scrotal incision was made, the testicles were visualized, and then testes were returned to the scrotal sac followed by skin closure as above. All animals received meloxicam 24 and 48 hours after the first injections to minimize post-operative pain. ORCH and SHAM mice were indistinguishable in their cages with no difference in body weight (Supplemental Figure 4A). Seminal vesicle weight, however, was markedly lower in ORCH mice, confirming the efficiency of androgen depletion (Supplemental Figure 4B). Colon lengths were 10% greater in ORCH mice compared to SHAM mice (Supplemental Figure 4C).

DHT subcutaneous implant procedure

Mice were anesthetized and provided with peri-procedural bupivacaine and meloxicam analgesia, as described above. Pellets containing 5-alpha-dihydrotestosterone (DHT) were implanted subcutaneously under sterile conditions (Innovative Research of America A-161). The pellets contained 7.5 mg DHT/pellet and were designed to continuously release 0.125 mg per day of DHT over 60 days. No post-procedure analgesia was administered, as advised by veterinary consultation.

Immunohistochemistry, microscopy, and cell quantification

Gut tissues were isolated and immunostaining performed as previously described (45). Primary antibodies used were: Human anti-ANNA-1 1:40,000 (Gift from V. Lennon), Rabbit anti-AR 1:250 (Santa Cruz sc-816), Rabbit anti-AR 1:1000 (Abcam ab133273), Chicken anti-GFP 1:1000 (AVES GFP-1020), Goat anti-SCFR 1:500 (R&D Systems AF1356), Rabbit anti-αSMA 1:200 (Abcam ab-5694), Rabbit anti-nNOS 1:1500 (ImmunoStar 24287), Rabbit anti-PGP9.5 1:1000 (Cedarlane CL95101), Rat anti-MHCII 1:1000 (EMD Millipore MABF33), Rabbit anti-S100b (DAKO GA504), Chicken anti-GFAP 1:1000 (Millipore AB5541), Mouse anti-CD3 17A2 1:500 (BioLegend 100201). Anti-goat and anti-donkey secondary antibodies used were conjugates of AlexaFluor 594, 568, or 488

(Invitrogen). Nuclei were counterstained with DAPI in Vectashield mounting medium (Vector Labs H-1200, Burlingame, CA). To quantify AR^+ neurons, 1.5cm segments of the colon were immunostained as whole mounts for ANNA-1 and AR. Single planar images were captured at the level of the myenteric plexus (6 fields per segment, identified based on ANNA-1 signal alone). Images were coded, randomized, and quantified using ImageJ by investigators blinded to the experimental condition. To quantify smooth muscle thickness in SHAM and ORCH mice, colons were acutely isolated, gently flushed to remove fecal pellets, and then fixed with 4% paraformaldehyde in phosphate buffered saline while cannulated on bamboo skewers, to ensure equivalent luminal distention across all samples. Cryosections of colonic tissue (14μm) were immunostained for SMA, mounted in Vectashield with DAPI, and imaged on an Olympus BX41 epifluorescent microscope. Twelve images were captured per animal and muscularis externa thickness was measured using SMA to mark boundaries. For human tissue analysis, IHC was performed on de-identified colon tissue sections obtained from the Columbia University Cancer Center Molecular Pathology Tissue Bank. Tissue sections derived from healthy margin tissue of resection specimens from patients 18-50 years of age. All images of immunohistochemical staining in the manuscript are representative of observations made in a minimum of 3 independent subjects per condition.

Gastrointestinal motility and stool composition analyses

Total gastrointestinal transit time, gastric emptying and small intestinal transit were measured as previously described (20). Fecal pellet output represents the number of pellets spontaneously excreted by each animal in 60-min. This and other in vivo motility tests were always initiated at 9am. For *ex vivo* imaging of colonic motility, colonic contractile activity was recorded and analyzed using Scribble and Matlab scripts, as previously described (17, 20). Briefly, this analysis generates spatiotemporal heat maps in which color represents gut width, the X-axis represents the proximal to distal length of the colon, and the Y-axis represents time. CMMCs were defined as contractions that originated in the

proximal colon and successfully propagated at least 50% of the length of the tissue. All parameters were measured from three 15-minute video recordings obtained from each of 6 mice per group, and all individual data points are shown in graphs. The organ bath preparation accommodates 2 colons at a time, and SHAM and ORCH colons were imaged in every session in parallel. To determine the rate and time required for relaxation of individual CMMCs, vertical slice analysis was performed at two positions along the x-axis of the heat map: one at $1/3^{rd}$ of the length of the colon (proximal slice) and one at 2/3rds of the length (distal slice). This generates line graphs at these two points showing gut width over time. The green lines shown in Figure 3F and 3H represent the vertical slices from the proximal positions and the pink lines represent the distal positions. From these graphs, the rate of relaxation was calculated from determining the change in gut width over the change in time as averaged from two contractions per video. For stool composition measurements, mice were transferred to clean cages without bedding at 9am and stool output was continuously collected for 1 hour. Animals had free access to both food and water during this time. Total mass of stool collected was "wet" weight per mouse. Fecal pellets were then dried at 45°C for a minimum of 48 hours until a stable mass was reached, and then a "dry" weight was recorded. Percent stool water content = [("wet"-"dry")/"wet"]*100). Average fecal pellet mass = "wet"/ # of pellets.

Smooth muscle contractility

Mice were euthanized with 40mg/kg sodium pentobarbital. The colons were removed and flushed with modified Krebs buffer (mM: NaCl 137, KCl 2.9, CaCl2 1.8, MgCl2 2.1, NaH2PO4 0.4, NaHCO3 11.9, D-Glucose 5.6, pH 7.4). Connective tissue was removed, and 3-4 mm lengths of transverse annular segments of the distal colon were excised ≥1 cm from the anus and massed. Each colonic ring segment was mounted in a chamber of a DMT 620M Multi-Wire Myograph (DMT, Ann Arbor, MI). The Krebs buffer was changed every 15 min (37°C, bubbled at 95% O2/ 5% CO2), and each tissue was equilibrated at 1 gram (g) resting tension for 1 hour. To demonstrate tissue viability, colonic

smooth muscle rings were isometrically contracted with three cycles of increasing log concentrations of acetylcholine (ACh) (10nM-1mM) (Sigma Aldrich, St. Louis, MO). Krebs buffer was exchanged, and tension was readjusted to 1g between each ACh cycle to re-establish the baseline tension. After the final ACh cycle, and following three buffer exchanges, all tissues were pretreated with 1uM tetrodotoxin (EMD Millipore, Jaffrey, NH) to mitigate the pro-relaxant effects of the myenteric nerve plexuses and maximize myogenic contractility. After 20 min pretreatment, the colonic rings were contracted with carbachol 1nM-4uM (Sigma Aldrich, St. Louis, MO) to define the EC50 carbachol of each colonic ring. The buffer was exchanged, and resting tension returned to 1g. Tetrodotoxin pretreatment was re-administered before maximal contraction was induced with 200mM KCl.

Gene expression analysis

Gut tissues from post-pubertal male and female FVB/NJ mice (n=5 mice per sex) were isolated, immediately transferred to TRIzol (Invitrogen), and stored at -80°C for processing. RNA was extracted using the standard phenol/chloroform method per manufacturer's instructions and purified using the RNeasy Micro Kit (Qiagen). Reverse transcription was performed with iScript cDNA Synthesis Kit (Bio-Rad Laboratories) and quantitative polymerase chain reaction (qPCR) was performed with SYBR Green Master Mix. Primers are: *Ar* Forward Primer (5'-3'): CTGGGAAGGGTCTACCCAC; *Ar* Reverse Primer (5'-3'): GGTGCTATGTTAGCGGCCTC; *Rpl19* Forward (5'-3'): ACCTGGATGAGAAGGATGAG; *Rpl19* Reverse (5'-3'): ACCTTCAGGTACAGGCTGTG. *Ar* expression was normalized to *Rpl19* expression and $2^{-\text{Delta CT}}$ was used to calculate gene expression changes across tissues. Data was analyzed using two-way ANOVA with multiple comparisons.

AR expression in human colonic myenteric ganglia was analyzed using a publicly available bulk RNA-sequencing dataset generated from samples obtained by laser capture microdissection (LCM; n = 6-8 samples per sex and 3 technical replicates per sample) (29). Raw data was downloaded from National Center for Biotechnology Information Gene Expression Omnibus under accession number: GSE153202.

Reads per kilobase per million-mapped Reads (RPKM) value of *AR* (ENSG00000169083) was extracted from this dataset and graphed by sex.

Statistical analyses of non-human data

For comparisons between pairs of means, unpaired Student's t-tests were used. All graphs display mean ± standard error of the mean (SEM) for each condition with each individual data point shown. "*n*" refers to number of mice per condition, except where specifically noted. A P value < 0.05 was considered significant and denoted with *. P values < 0.005 are noted with ** and < 0.0005 with ***. For comparisons of means from 3 of more groups, ANOVA was used. For repeated measures over time compared between two groups, 2-way ANOVA and post-hoc Tukey tests were performed. For muscle contractility measurements, muscle force values were normalized to each colonic ring's mass. Sigmoidal dose-response curves were fitted with nonlinear regression, and best-fit parameters (logEC50, top bound, expressed as value ± SEM) between groups were compared using extra sum-of-squares F test in Prism 4.0 (Graphpad, San Diego, CA).

Human IBS study and statistical analysis

Androgen and sex hormone binding globulin (SHBG) levels were measured in serum collected at the baseline visit of a randomized controlled trial designed to evaluate open label placebo as a therapeutic modality in patients with IBS (Clinical Trials.gov NCT02802241). All subjects in this IRB-approved trial were recruited from a single academic medical center in the United States of America. Written informed consent was obtained from all subjects. Hormone measurements were obtained using liquid chromatography tandem mass spectrometry (LC-MS/MS) assays and SHBG was measured using a chemiluminescence immunoassay. Assays were performed by the Brigham Research Assay Core (BRAC; Brigham and Women's Hospital, Boston, MA), which is certified by the Center for Disease Control's Hormone Assay Standardization Program (HoST). Measurements were made for all 209 individuals with IBS in the study for whom serum samples were available from the baseline visit and 28

healthy controls (HC). Of these, one individual with IBS was excluded due to an indeterminate measurement. Final analyses were based on the remaining 236 subjects (208 IBS, 28 HC) using SPSS (IBM SPSS Statistics version 27, IBM Corp., Armonk, NY, U.S.A.). Supplemental Table 1 reports descriptive data of study subjects, including age and distribution of IBS-subtypes, as means and standard deviations (SD). This study was not powered to detect subtype-specific differences in hormone levels. Median values and interquartile ranges (IQR) of all hormone levels are reported in Supplemental Table 2, and distributions are illustrated as violin plots in Figure 1, A and B and Supplemental Figure 7. Because the distributions of the hormone data were skewed, statistical analysis was conducted after transforming this data using a logarithmic scale (with natural log values). This transformation was applied to all measurements except for percent free testosterone because this measure was already normally distributed. T-tests were used to evaluate the differences in mean log-transformed levels between IBS and HC subjects, separately for males and females.

The Irritable Bowel Syndrome-Symptom Severity Scale (IBS-SSS) is a validated 5-question survey used to generate a composite score based on: severity of abdominal pain, number of days with abdominal pain over the preceding 10 days, presence and severity of abdominal distension, satisfaction with bowel habits, and IBS-related quality of life (14). Each of these 5 components is scored on a scale of 1-100 with a maximum composite score of 500. Partial correlation analysis was used to assess the association between IBS-SSS and percent free testosterone. Student’s t-test revealed no difference in mean percent free testosterone between male and female IBS patients, so the groups were combined for correlation analysis to increase power, controlling for age and sex (results shown in Figure 1C).

Supplemental Data

Table S1. Characteristics of human study participants.

Table S2. Diminished testosterone levels are associated with the diagnosis of IBS.

Figure S1. Gonadal androgen deficiency disrupts colonic motility.

Figure S2. Androgen receptor (*Ar*) transcripts are present at comparable levels along the entire length of the male and female gastrointestinal tract in mice.

Figure S3. Gonadal androgen deficiency does not grossly alter populations of enteric glia, immune cells or interstitial cells of Cajal in the colon.

Figure S4. Loss of gonadal function alters androgen-responsive tissues but not body weight or enteric neuronal density.

Figure S5. Wnt1-Cre2 transgenic mice exhibit Cre activity in enteric neurons, but not smooth muscle, in the adult mouse colon.

Figure S6. Conditional genetic depletion of androgen signaling in the peripheral nervous system.

Figure S7. Distribution of percent free testosterone and sex hormone binding globulin (SHBG) levels in IBS patients and healthy controls.

Movie S1. Colonic contractile activity is disorganized and less effective at oral-to-anal propulsion of luminal contents in male mice lacking gonadal function.

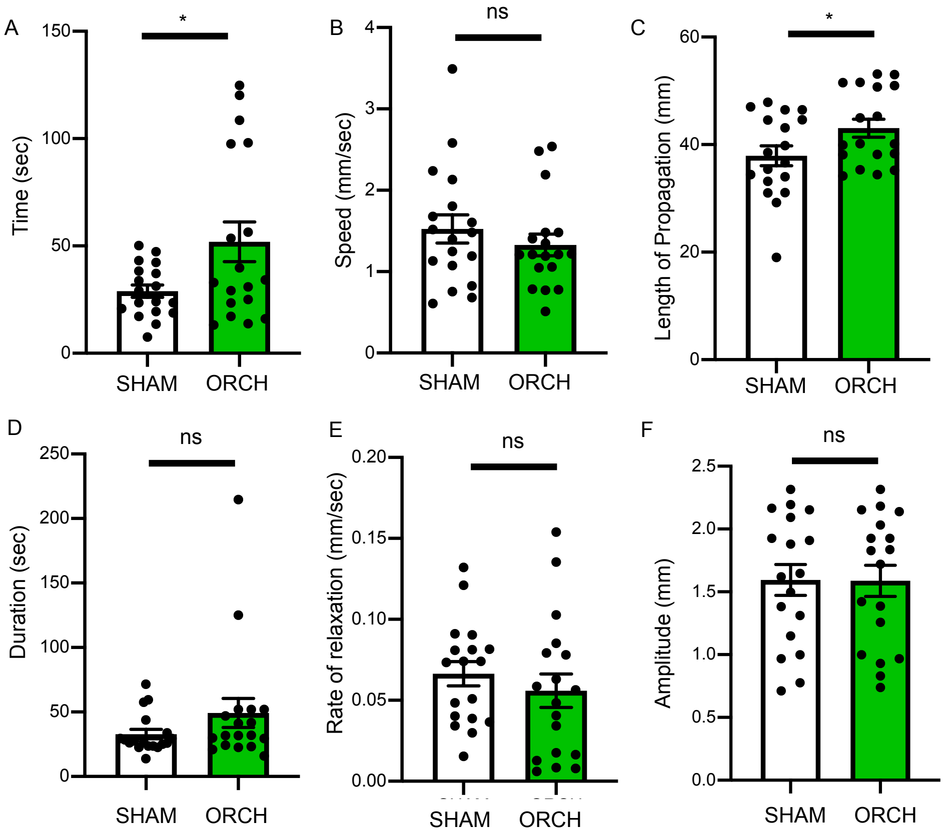

Supplemental Figure 1. Gonadal androgen deficiency disrupts colonic motility.
Features of colonic migrating motor contractions (CMMCs) in colons from SHAM and ORCH mice that were acutely isolated and imaged *ex vivo*, four or more weeks after surgery. CMMCs were defined as contractions that originated in the proximal colon and successfully propagated at least 50% of the length of the colon. All parameters were measured from three 15-minute video recordings obtained from each of 6 mice per group. Each individual data point is shown. The organ bath preparation accommodates 2 colons at a time, and SHAM and ORCH colons were imaged in every session in parallel. *P*-values reflect unpaired t-tests and error bars reflect standard error of the mean. * Denotes $P < 0.05$. ns denotes $P > 0.05$.

A. Time in seconds (sec) required for colons to relax from maximal contraction to baseline gut width following a CMMC was greater in colons from ORCH mice ($P = 0.034$).

B. CMMC speed was no different between colons from SHAM and ORCH mice ($P = 0.3755$).

C. Length of propagation of individual CMMCs was greater in colons from ORCH mice ($P = 0.0458$), compared to SHAM controls, proportional to the longer colonic length in ORCH mice (see Supplemental Figure 5C).

D. Duration of CMMCs was no different between colons from SHAM and ORCH mice ($P = 0.1740$).

E. Rate of colonic relaxation following a CMMC was no different in colons from SHAM and ORCH mice ($P = 0.4152$).

F. Amplitude of gut contraction during CMMCs was no different in colons from SHAM and ORCH mice ($P = 0.9717$).

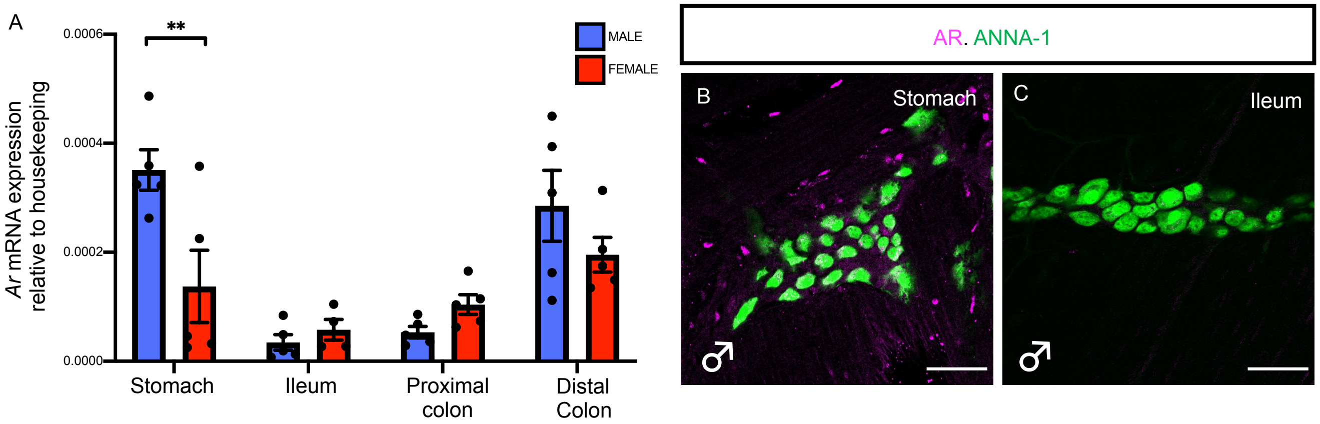

Supplemental Figure 2. Androgen receptor (*Ar*) transcripts are present at comparable levels along the entire length of the male and female gastrointestinal tract in mice.

A. *Ar* mRNA levels in various regions of the GI tract in post-pubertal males and females, measured by quantitative RT-PCR relative to the housekeeping gene *Rpl19*. Two-way ANOVA with multiple comparisons found significant difference in *Ar* expression between males and females only in the stomach (n= 5 mice per sex per region).

B and **C.** AR immunoreactivity is detectable in the muscularis externa of the stomach (**B**) but not small intestine (**C**) of an adult male mouse. In the stomach, AR immunoreactivity does not colocalize with the pan-neuronal marker (ANNA-1).

Scale bars in **B** and **C** = 50µm.

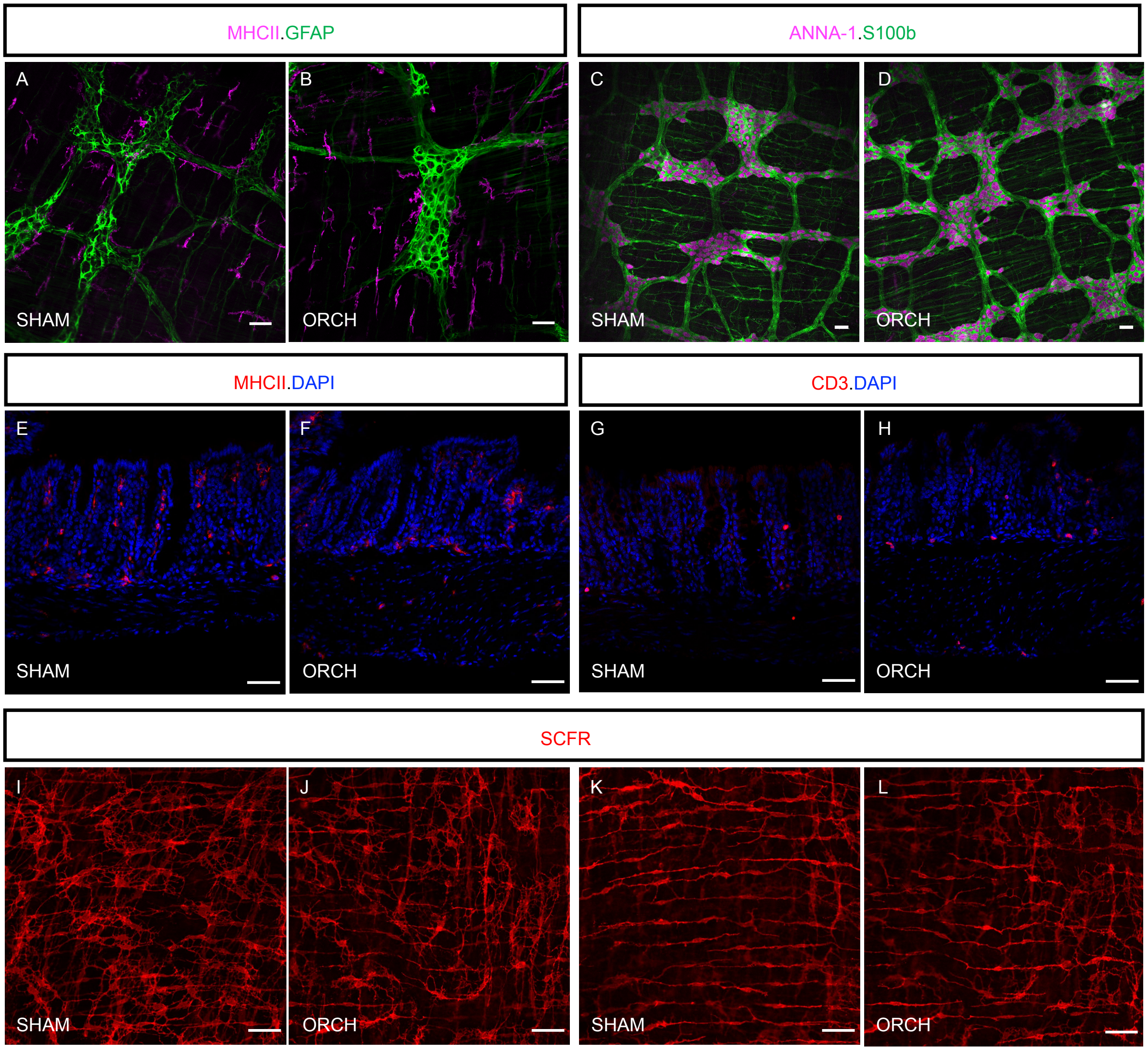

Supplemental Figure 3. Gonadal androgen deficiency does not grossly alter populations of enteric glia, immune cells or interstitial cells of Cajal in the colon.

A and **B.** Whole mounts of colons from SHAM and ORCH mice immunostained for MHCII (to label muscularis macrophages) and GFAP (to label enteric glia) and imaged at the level of the myenteric plexus revealed no differences.

C and **D.** Whole mounts of colons from SHAM and ORCH mice immunostained for ANNA-1 (to label enteric neurons) and S100B (to label enteric glia) and imaged at the level of the myenteric plexus revealed no differences.

E and **F.** Cross-sections of colons from SHAM and ORCH mice immunostained for MHCII show no major differences in macrophage numbers or localization.

G and **H.** Cross-sections of colons from SHAM and ORCH mice immunostained for CD3 to label T lymphocyte cells show no major differences in T-cell infiltrates.

E - H Cell nuclei marked with DAPI.

I - L. Whole mounts of colons from SHAM and ORCH mice immunostained for stem cell factor receptor (SCFR), a marker for interstitial cells of Cajal (ICC), and imaged at the level of the myenteric plexus (**I, J)** and circular muscle (**K, L**) revealed no major difference in ICC networks. Panels **J** and **L** represent the same field of view of the same tissue specimen imaged at two different z-planes (optical sections) to visualize the two types of ICC networks in the same colon.

Scale bars in **A – L** = 50µm. All images are representative of observations made in a minimum of 3 mice per condition.

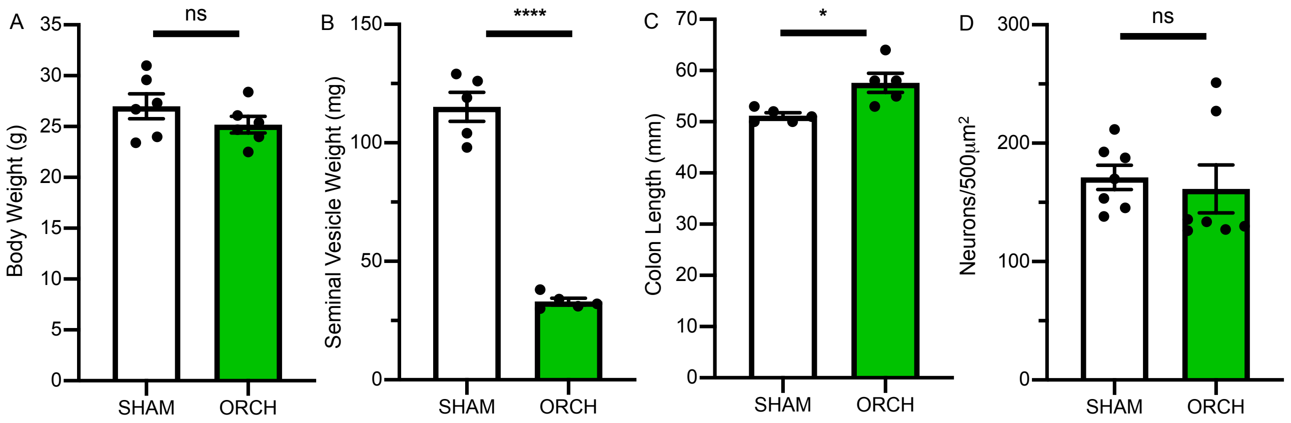

Supplemental Figure 4. Loss of gonadal function alters androgen-responsive tissues but not body weight or enteric neuronal density.

A. Body mass measured in 10 week old mice, four weeks after bilateral orchiectomy (ORCH) or sham surgery (SHAM), was no different between the groups (*N* = 6 mice/group).

B. Mass of seminal vesicles, a tissue well-known to atrophy in the absence of androgens, is markedly lower in ORCH than SHAM mice 4 weeks after surgery (*N* = 5 mice/group).

C. Colon lengths in ORCH mice are 10% greater compared to SHAM mice, 4-6 weeks after surgery (*N* = 5 mice/group).

D. Neuronal density in the myenteric plexus, measured by whole mount immunostaining of colonic segments from SHAM and ORCH mice for ANNA-1, shows that loss of gonadal function does not alter enteric neuronal number five weeks after surgery (*N* = 7 mice/group). Unpaired t-tests were used to compare pairs of group means. Error bars reflect standard error of the mean. * Represents $P < 0.05$, ** $P < 0.01$, *** $P < 0.005$, and **** $P < 0.001$.

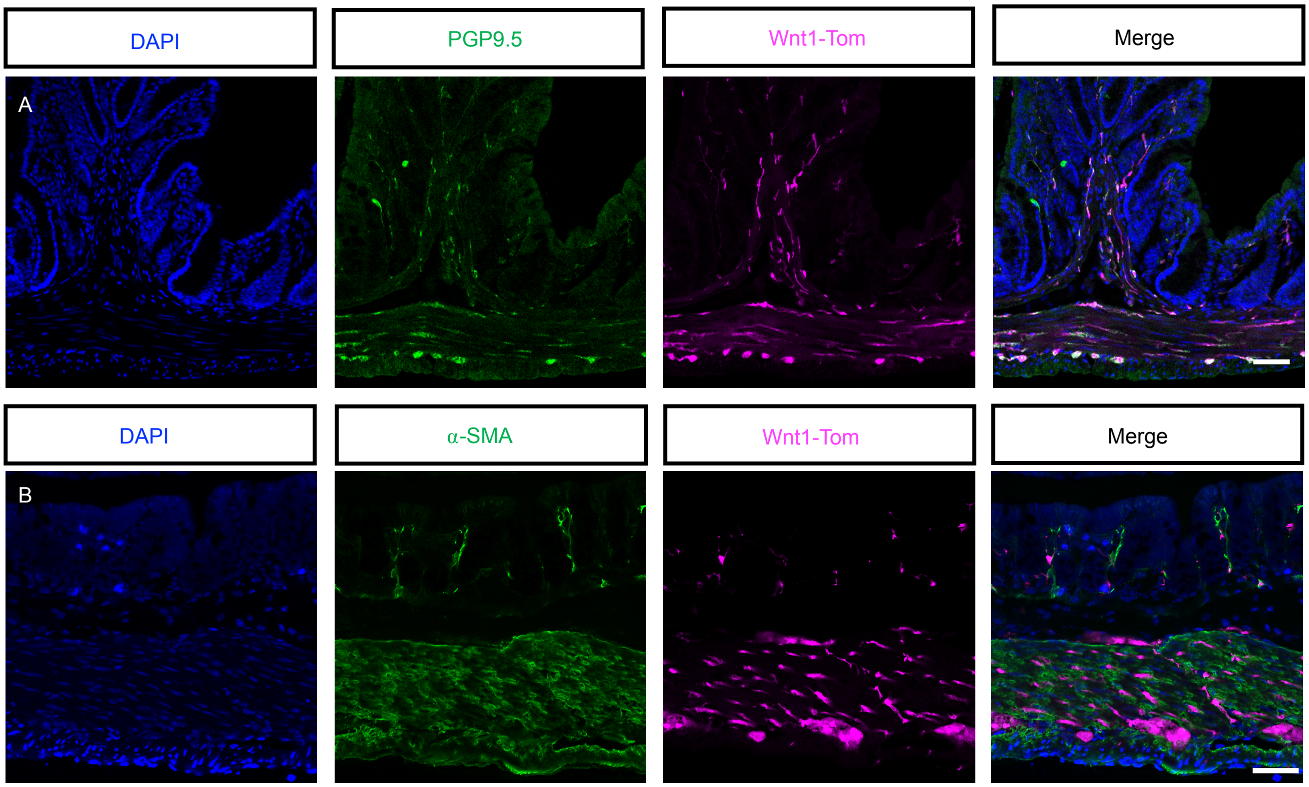

Supplemental Figure 5. Wnt1-Cre2 transgenic mice exhibit Cre activity in enteric neurons, but not smooth muscle, in the adult mouse colon.
$Wnt1^{Cre2}$::$Rosa26^{Ai9/+}$ mice were analyzed to assess Cre activity in the colon. Cre activity induces expression of a tdTomato fluorescent reporter protein in Ai9 mice. Representative cross-sections of colons obtained from 10-week old mice are shown. Cross-sections immunostained for the pan-neuronal marker PGP9.5 (panels in row **A**) show that PGP9.5 colocalizes with the tdTomato reporter, confirming Cre activity in the majority of enteric neurons. In contrast, the smooth muscle marker a-smooth muscle actin (SMA) does not colocalize with tdTomato (panels in row **B**), indicating that there is no Cre-mediated recombination in the colonic smooth muscle. Nuclei counterstained with DAPI. Scale bars = 50µm. Images are representative of observations made in a minimum of 3 mice per condition.

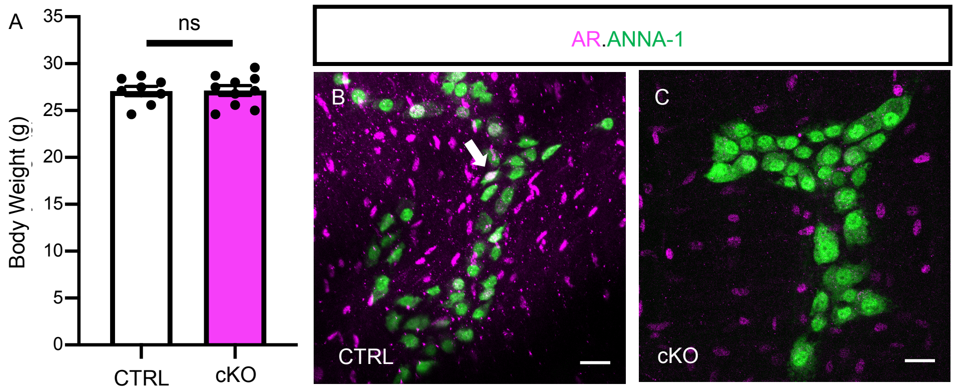

Supplemental Figure 6. Conditional genetic depletion of androgen signaling in the peripheral nervous system.
A. Body mass measured in 10 week old AR^{Wnt1KO} mice (cKO) and littermate controls (CTRL; $Ar^{flx/Y}$) was no different between the groups (*N* = 8-10 mice/group). "ns" represents *P* = 0.9545 resulting from unpaired t-test comparing group means. Error bars reflect standard error of the mean.
B, C. Androgen receptor (AR) and pan-neuronal marker (ANNA-1) immunoreactivities show that AR expression is evident in both myenteric neurons and surrounding smooth muscle in colons from 10 week-old $AR^{flx/Y}$ control mice (CTRL), but undetectable in enteric neurons of AR^{Wnt1KO} mice (cKO). Arrow marks an AR^+ neuron in the CTRL colon. Scale bars = 25 µm. Images are representative of observations made in a minimum of 3 mice per condition.

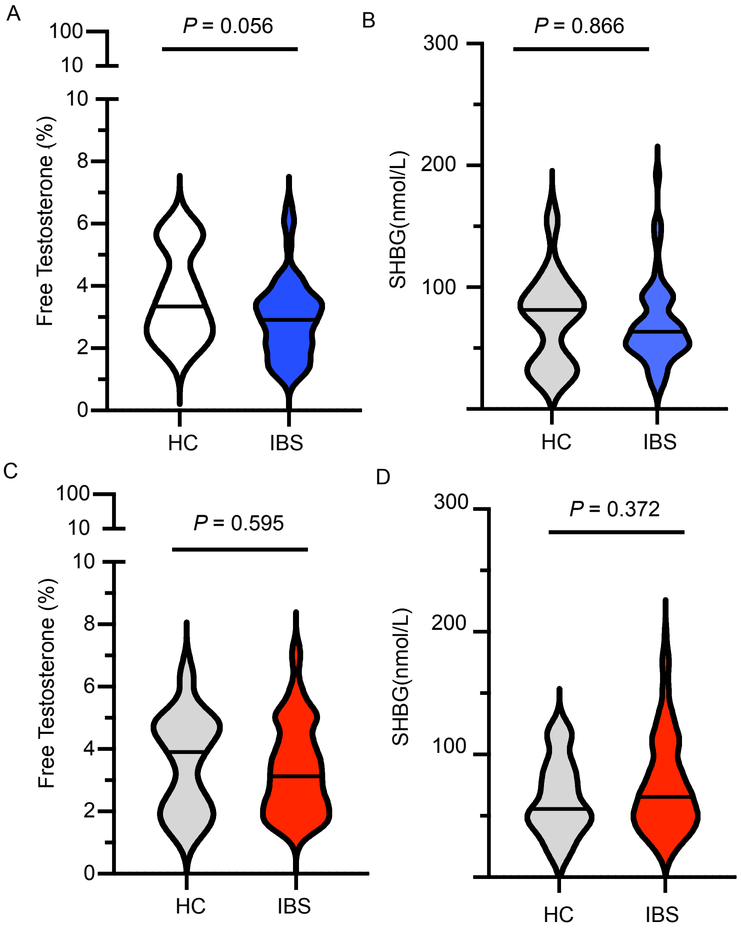

Supplemental Figure 7. Distribution of percent free testosterone and sex hormone binding globulin levels among IBS patients and healthy controls.

Violin-plots illustrating levels of percent free testosterone and sex hormone binding globulin (SHBG) in healthy controls (HC) and patients with IBS. Median value is indicated as bar in center of each plot. P values are for unpaired t-tests comparing means (% free testosterone) or means of log-transformed values (SHBG) between IBS and HC groups.

A and **B.** Males

C and **D.** Females

	MEAN AGE	SD	MEAN IBS-SSS	SD
Male				
IBS-All Subtypes (58)	43.19	17.29	280.36	66.30
IBS-C (14)	50.43	22.84	273.07	65.29
IBS-D (41)	41.93	14.69	278.49	67.23
IBS-M (1)	27.00	.	374.00	.
IBS-U (2)	26.50	2.12	323.00	32.53
Healthy Controls (14)	34.79	10.65	-	-
Female				
IBS-All Subtypes (150)	43.34	19.16	271.28	63.69
IBS-C (59)	40.64	18.14	285.12	65.45
IBS-D (66)	46.77	20.50	258.62	60.73
IBS-M (22)	42.23	17.32	276.09	59.73
IBS-U (3)	29.00	8.72	242.33	90.18
Healthy Controls (14)	38.64	13.74	-	-

Supplemental Table 1. Characteristics of human study participants.
Mean age, mean IBS symptom severity score (IBS-SSS) and associated standard deviations (SD) for healthy control study participants, and participants with IBS categorized into individual IBS subtypes. IBS-Constipation (IBS-C), IBS-Diarrhea (IBS-D), IBS-Mixed presentation (IBS-M), and IBS-Undetermined (IBS-U).

	IBS		HC		
	MEDIAN	IQR	MEDIAN	IQR	P-value
Male	(N=58)		(N=14)		
Total Testosterone (ng/dL)	451.00	282.00	618.00	230.50	0.106
% Free Testosterone	2.91	1.55	3.35	2.92	0.056
Absolute Free Testosterone (ng/dL)	11.86	5.53	17.38	14.72	**0.002**
SHBG (nmol/L)	31.72	18.96	40.73	30.79	0.866
DHT (ng/dL)	38.70	32.50	36.35	43.37	0.324
Female	(N=150)		(N=14)		
Total Testosterone (ng/dL)	17.40	12.25	29.60	12.68	**0.001**
% Free Testosterone	3.13	2.23	3.91	2.80	0.595
Absolute Free Testosterone (ng/dL)	0.58	0.23	0.85	0.84	**<0.0001**
SHBG (nmol/L)	65.58	52.98	55.71	45.13	0.372
DHT (ng/dL)	4.52	4.28	5.73	2.97	0.103

Supplemental Table 2. Diminished testosterone levels are associated with the diagnosis of IBS.
Median values and inter-quartile ranges (IQR) for total testosterone, percent (%) free testosterone, sex hormone-binding globulin (SHBG), and dihydrotestosterone (DHT) measured in the sera of a cohort of post-pubertal adults with irritable bowel syndrome (IBS) and healthy controls (HC). Absolute free testosterone was calculated by multiplying total testosterone by the percent that was free. N = number of subjects. P-values are for t-tests comparing means (% free testosterone) or means of log-transformed values (all others) between IBS and HC groups of each sex.

Supplemental Video 1. Colonic contractile activity is disorganized and less effective at oral-to-anal propulsion of luminal contents in male mice lacking gonadal function.
Video-recordings of motor activity in colons from 10-week old mice four weeks after sham operation (SHAM, top bath) and bilateral orchiectomy (ORCH, bottom bath). Colons are oriented with oral end on the left and anal end on the right. The colon from the SHAM mouse shows distinct colonic migrating motor contractions (CMMCs) that progress from oral to anal ends of the colon, as typically seen in colons from healthy, adult mice. The colon from the ORCH mouse, in contrast, has numerous disorganized, irregular contractions that do not consistently progress from oral to anal ends. These recordings are representative of observations made in three 15-minute videos obtained from each of at least 5 mice per condition.
